# Design feature combinations effects of running shoe on plantar pressure during heel landing: A finite element analysis with Taguchi optimization approach

**DOI:** 10.3389/fbioe.2022.959842

**Published:** 2022-09-13

**Authors:** Zihan Yang, Chuyi Cui, Xianglin Wan, Zhiyi Zheng, Songhua Yan, Hui Liu, Feng Qu, Kuan Zhang

**Affiliations:** ^1^ School of Biomedical Engineering, Capital Medical University, Beijing, China; ^2^ Beijing Key Laboratory of Fundamental Research on Biomechanics in Clinical Application, Capital Medical University, Beijing, China; ^3^ School of Sport Sciences, Beijing Sport University, West Lafayette, IN, United States; ^4^ Fashion Accessory Art and Engineering College, Beijing Institute Of Fashion Technology, Beijing, China; ^5^ College of Health and Human Sciences, Purdue University, West Lafayette, IN, United States; ^6^ Anta Sports Science Laboratory, Xiamen, China; ^7^ China Institute of Sport and Health Science, Beijing Sport University, Beijing, China

**Keywords:** rearfoot strike, shoe design, plantar pressure, finite element method, Taguchi method

## Abstract

Large and repeated impacts on the heel during running are among the primary reasons behind runners’ injuries. Reducing plantar pressure can be conducive to reducing running injury and improving running performance and is primarily achieved by modifying the design parameters of running shoes. This study examines the effect of design parameters of running shoes (i.e., heel-cup, insole material, midsole material, and insole thickness) on landing peak plantar pressure and determines the combination of different parameters that optimize cushion effects by employing the Taguchi method. We developed the foot–shoe finite element (FE) model through reverse engineering. Model assembly with different design parameters was generated in accordance with the Taguchi method orthogonal table. The effectiveness of the model was verified using the static standing model in Ansys. The significance and contribution of different design parameters, and the optimal design to reduce plantar pressure during landing, were determined using the Taguchi method. In the descending order of percentage contribution was a conforming heel-cup (53.18%), insole material (25.89%), midsole material (7.81%), and insole thickness (2.69%). The more conforming heel-cup (*p* < 0.001) and softer insole (*p* = 0.001) reduced the heel pressure during landing impact. The optimal design of running shoe in this study was achieved with a latex insole, a 6 mm insole thickness, an Asker C-45 hardness midsole, and a 100% conforming heel-cup. The conforming heel-cup and the insole material significantly affected the peak plantar pressure during heel landing. The implementation of a custom conforming heel-cup is imperative for relieving high plantar pressure for long-distance heel-strike runners.

## Introduction

Between 37 and 56% of runners suffer running injuries at least once a year (Van Mechelen, 1992), frequently resulting from hard landings and repeated impact (Torg et al., 1974; Andréasson and Peterson, 1986; Pine, 1991; Nigg and Wakeling, 2001). Over 75% of long-distance runners primarily perform heel landing when running at a medium speed (Kerr, 1983; Hasegawa et al., 2007). Rapid deceleration following heel contact exerts significant instantaneous ground reaction forces (GRFs) on the heel (Denoth, 1986), which lead to the increased risk of injuries compared to forefoot running (Sanzén et al., 1986; Willems et al., 2006; Song et al., 2013). It has been demonstrated that excessive impact force is predictive of a wide variety of running injuries (Milner et al., 2006; Pohl et al., 2009; Zadpoor and Nikooyan, 2011; Bredeweg et al., 2013; Chan et al., 2018). Therefore, reducing the impact load through the redistribution of plantar foot pressure has been an important target during the design of running shoes.

To redistribute plantar foot pressure (Cheung and Zhang, 2005; Peng et al., 2022), several shoe parameters were modified in terms of their shape and material of the midsole and insole (Actis et al., 2008; Antunes et al., 2008; Cheung and Zhang, 2008). Conventional methods of determining these parameters required an extensive research and development cycle. The finite element (FE) method facilitated the efficient evaluation of different designs and material parameters of the running shoe without the prerequisite of fabricating it and replicating subject trials (Cheung and Zhang, 2008), representing a time- and labor-saving solution to the cycle (Hannah et al., 2016; Peng et al., 2022). Previous FE analyses indicated that the arch-conforming shape (Cheung and Zhang, 2008; Peng et al., 2022), insole thickness (Chatzistergos et al., 2015), insole and midsole material (Cheung and Zhang, 2008; Chatzistergos et al., 2015; Peng et al., 2022), and insole lateral wedge angle (Peng et al., 2022) were conducive to reducing the plantar pressure during static standing. While some studies investigated the shoe design factors on plantar pressure during standing, their contribution to reducing heel impact forces during running remains unclear.

Previous studies on the plantar pressure distribution of landing the foot during running employed either a vertical landing model or a partial foot and insole model. However, the impact area and foot kinematics of heel landing during running differ significantly from static standing or vertical landing. Therefore, models for vertical landing (Goske et al., 2006; Cho et al., 2009) or an incomplete FE model (Goske et al., 2006; Fontanella et al., 2013; Hannah et al., 2016; Drougkas et al., 2018) limit the accuracy of simulation results and systematic investigation of the heel impact in response to various running design combinations. To the best of our knowledge, this is the first study to explore plantar pressure with a complete foot–shoe FE model during heel landing. The complexity of the FE foot–shoe model limits the study of heel landing. Several simplified or incomplete FE models were proposed for studying heel landing. For instance, grounded midsole (Fontanella et al., 2013; Nonogawa et al., 2020) or midsole foot bonding (Hannah et al., 2016) provides different boundary conditions for landing in shoes. Hence, a complete foot–shoe FE model for authentic shoes available for purchase is required to effectively simulate heel impact.

The evaluation of different multiple factor designs and level combinations for running shoes can be time-consuming and costly with conventional testing methods (Rao et al., 2008). A multi-factor and multi-level experimental method in accordance with the orthogonal array, known as the Taguchi method, is a statistical method for FE analysis (Dar et al., 2002; Rao et al., 2008). This allows a balanced comparison of levels of any factor with less effort (Cheung and Zhang, 2008), and has been extensively used to investigate the sensitivity of the design parameters in FE models (Cheung and Zhang, 2008; Zhang et al., 2020; Peng et al., 2022).

The aim of the present study was to determine how to lower the peak plantar heel pressure with different shoe parameter combinations based on a complete foot–shoe FE model developed through reverse engineering (Jeng et al., 2012) and the Taguchi method. We hypothesized that all design parameters significantly affect the peak plantar pressure and that there exists an optimal combination to minimize it.

## Materials and methods

**FIGURE 1:**
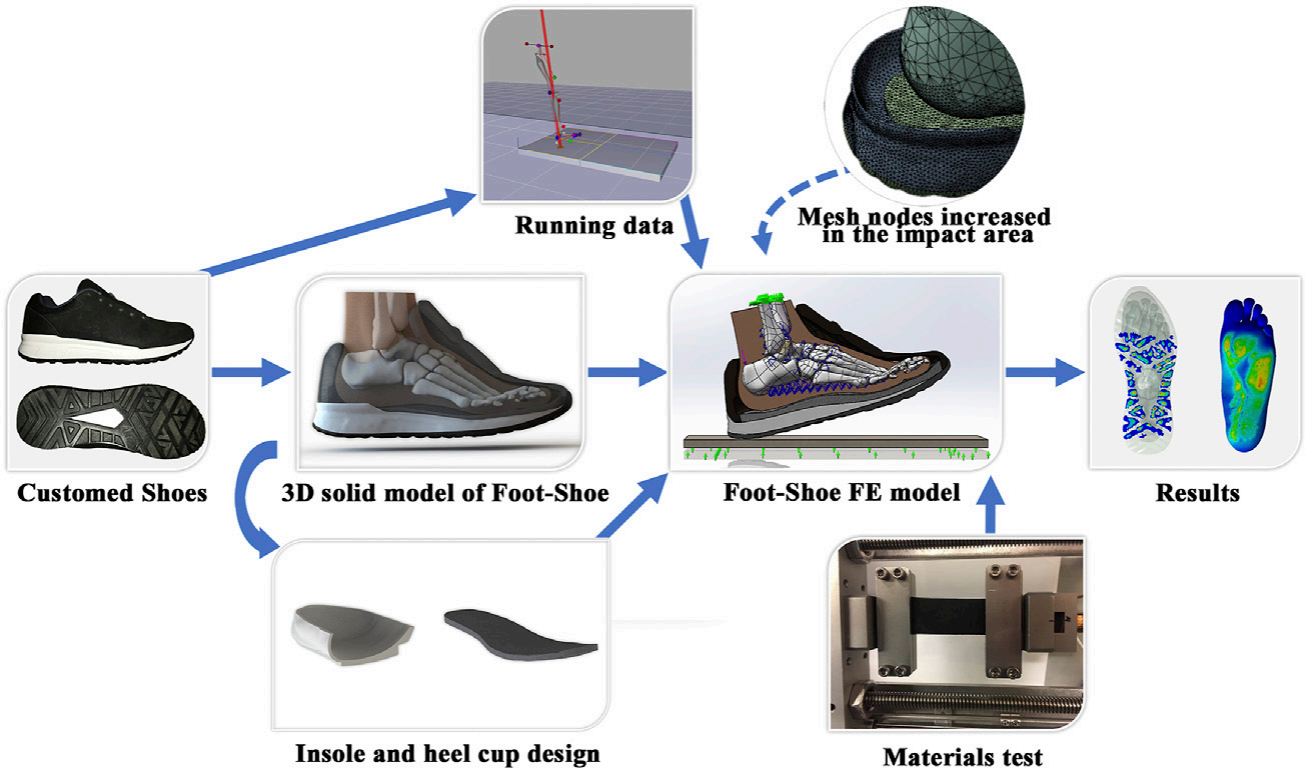
Overview of the foot–shoe FE model. The 3D solid model of foot–shoe is based on custom shoes. Different insole and heel-cups were designed in SOLIDWORKS, and various midsole and insole materials were tested. Kinematic and kinetic running data were collected and input as boundary conditions, and mesh nodes in the impact area were increased to obtain smooth prediction results.

To simulate the rearfoot strike in running and estimate the effect of different shoe design parameters on plantar pressure, we scanned the foot and shoe from a single individual and constructed the FE model through reverse engineering. We set the boundary conditions using running kinematics and kinetic data from the model participant. Finally, we employed the statistics-based Taguchi method (Peng et al., 2022) to investigate the effect of different design feature combinations of running shoes on plantar pressure, including conforming heel-cup, insole material, midsole material, and insole thickness. The workflow is depicted in Figure 1.

### Reverse engineering data acquisition

A foot–shoe CT scan was obtained from a model participant (25 year old, male, height: 168 cm, weight: 67 kg) wearing custom running shoes (size: US 41, insole and midsole material: ethylene-vinyl acetate (EVA), insole hardness: Asker C-45, midsole hardness: Asker C-60, outsole material: rubber). This study was approved by the Institutional Review Board of Beijing Sport University (Number: 2021176H). CT images were scanned at 1.25-mm intervals from the participant’s right foot in a weight-off position.

### Kinematic and kinetic data acquisition

The participant performed five heel-strike running trials while wearing the shoes on a 20-m runway. The running speed was maintained at 3.8 ± 0.2 m/s using a timing system (300-series, Newtest oy, Oulu, Finland). Four force plates (9281CA, Kistler, Zürich, Switzerland) were embedded in the middle of the runway, sampling at 1000 Hz. Foot contact with the ground occurred within one of the force plates for all five trials. Lower limb kinematics were tracked with 19 retroreflective markers based on the modified Helen Hayes model (Vaughan et al., 1999) at 200 Hz using an eight-camera motion capture system (Raptor-4, Motion analysis, Rohnert Park, CA, United States). Gait biomechanical variables (e.g., GRF, foot–ground angle, net ankle joint force, ankle dorsiflexion moment) were obtained at the instant of vertical impact peak force for each trial (Willems et al., 2005). Plantar and outsole pressures of natural standing were collected synchronously using Footscan (V9 essentials, Rsscan, Beringen, Belgium) and Pedar Insole (Pedar-x, novel, Munich, Germany).

### Material properties

The material properties of shoe midsole and insole materials were tested, using custom size EVA and Latex materials. Three separate EVA materials (7.5 cm long, 2 cm wide, 5 mm thick) with different hardnesses (Asker C-45, 60, and 70) were tested using a MicroTester (eXpert 4000, ADMET, Norwood, MA, United States). The latex material (7.5 cm long, 2 cm wide, and 1 cm thick) was tested using a push–pull tester (HPH, Handpi LLC, Yueqing, Zhejiang, China). The respective material was tested three times, and the results of uniaxial tension with a testing speed of 1 mm/s and strain were recorded. The stress–strain curve and Poisson’s ratio were obtained for each tested material. The stress–strain curve was used as input parameters for Mooney–Rivlin or Ogden hyper-elastic material models simulated in the FE analysis (Table 1).

**TABLE 1:** Material properties in FE model.

Part name	Young’s modulus (MPa)	Poisson’s ratio	Element number	Element type	References
Encapsulated soft tissue	0.15	0.45	129357		Hsu et al. (2008)
Bone	7300	0.3	80705		Cheung and Zhang (2008)
Cartilage	10	0.4	229174		Li et al. (2018)
Upper shoe	11.76	0.35	16557		Cho et al. (2009), Li et al. (2018)
Outsole	8	0.47	24069		Huang et al. (2018)
Midsole Asker C-45	3 parameters Mooney–Rivlin model (C_10_ = −0.340138, C_01_ = 0.925877, C_11_ = 0.061484)	0.375	42090		
Midsole Asker C-60	3 parameters Mooney–Rivlin model (C_10_ = −1.41284, C_01_ = 2.61711, C_11_ = 0.203587)	0.375	42090	3D tetrahedral (C3D10)	
Midsole Asker C-70	3 parameters Mooney–Rivlin model (C_10_ = −2.09659, C_01_ = 3.65544, C_11_ = 0.327862)	0.375	42090		
EVA insole	3 parameters Mooney–Rivlin model (C_10_ = −0.340138, C_01_ = 0.925877, C_11_ = 0.061484)	0.375	13436-16589		
Latex insole	1st-order Ogden model (μ = 0.69787 MPa, α= 2.57)	0.4	13436-16589		
EVA heel cup	3 parameters Mooney–Rivlin model (C_10_ = −0.340138, C_01_ = 0.925877, C_11_ = 0.061484)	0.375	22266-48514		
Latex heel cup	1st-order Ogden model (μ = 0.69787 MPa, α= 2.57)	0.4	22266-48514		
Plantar fascia	350	-	-	Tension-only Truss	Cheung et al. (2006)
Ground plate	17000	0.1	1613	3D Brick (S8R)	Cheung and Zhang (2008)

### Full foot–shoe finite element model construction

To build the foot–shoe structural model, we reconstructed the foot (bones, encapsulated soft tissues) and shoe geometries (upper, insole, midsole, and outsole) from the CT scans in Mimics (Version 10.0, Materialise, Leuven, Belgium), smoothed in Geomagic (Version 2015, 3D Systems, Research Triangle Park, NC, United States) and assembled in SOLIDWORKS (Version 2018; SolidWorks Corp., Waltham, MA, United States).

Because cartilage and ligaments cannot be recognized from CT scans, cartilage was rebuilt in SOLIDWORKS based on anatomical locations (Human Anatomy Atlas, Visible Body, Newton, MA, United States). The middle and distal ends of the fifth metatarsal were fused because the latter was relatively small. The different insole and heel-cup shapes were designed in SOLIDWORKS, and the heel-cup and the insole were segmented, whereas the materials used for the heel-cup and the insole in one analysis remained unchanged. Lastly, a ground plate was built into the FE model to simulate ground support (Figure 1).

Similarly, foot ligaments (including the plantar fascial) were simulated using tension-only springs, in Ansys (Version 19.0, ANSYS, Canonsburg, PA, United States), and the resultant stiffness data were between 39.1 and 650 N/mm (Siegler et al., 1988; Cheung et al., 2006; Hoefnagels et al., 2007). Table 1 lists the part and element information in the foot–shoe model (Table 1). We used the bonded contact between the bone, encapsulated soft tissues, and cartilage elements, thus adding frictional contact to potential contact surfaces with cavities (friction coefficient μ = 0.6) (Cheung et al., 2005).

To increase the accuracy of the analysis and obtain higher solution convergence, a local mesh refinement was carried out on the heel region to accommodate the simulation of heel impact. The remaining components were automatically generated using the software in accordance with size. The mesh size of the ground plate was 5 mm. The mesh size of the feet and shoes was 2–10 mm following refinement.

**FIGURE 2:**
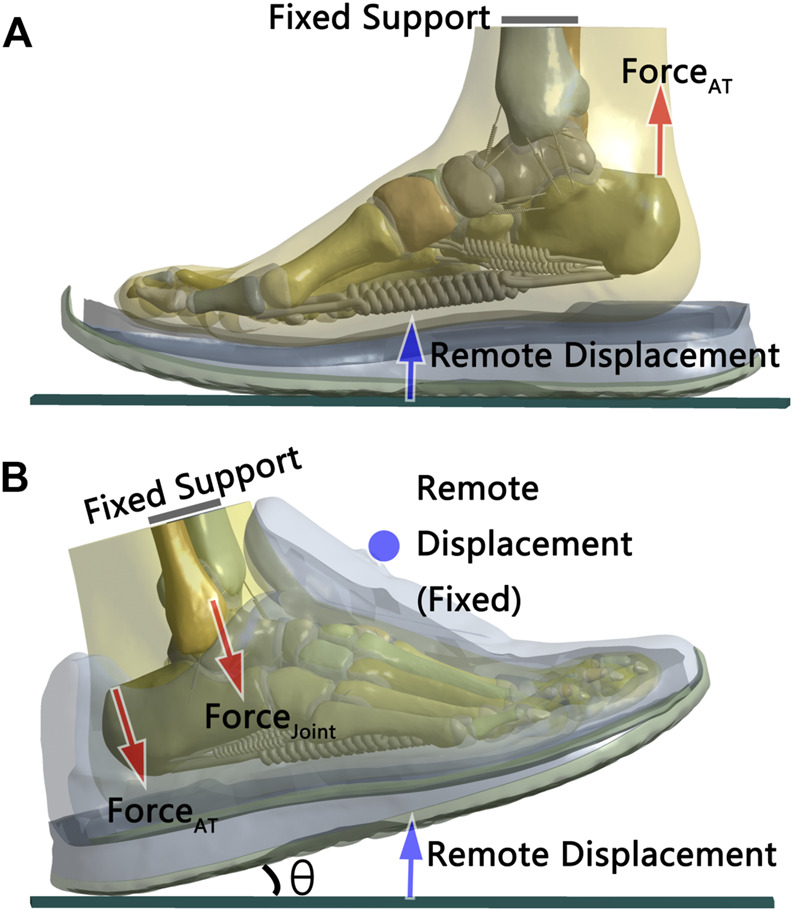
FE model and boundary set-up for **(A)** static standing and **(B)** rearfoot striking. “θ” represented the foot strike angle between the shoe model and ground plate.

The final foot model consisted of 29 bony segments, 69 ligaments, and 15 cartilage segments embedded in a volume of encapsulated soft tissues. The shoe model consisted of an upper, a heel cup, insole, midsole, and an outsole (Figure 2). The entirety of the tetrahedral elements was present in our model with acceptable quality in mesh control. The skewness value was 0.36 ± 0.21, where 0 corresponds to an equilateral tetrahedral element (Ansys, 2013). The aspect ratio was 2.22 ± 1.37, which represents relatively good mesh quality (Drougkas et al., 2018).

### Static stand load and boundary conditions

Our FE model was validated through static standing (i.e., validation model; Figure 2A). To simulate standing and landing, nonlinear large deformation quasi-static contact analysis was employed in this model. The shoe’s upper was removed to simplify the static stand model calculation. Bonded contact was used between the insole and midsole and between the midsole and outsole. Frictional contact (μ = 0.6) was used between the foot and insole and between the outsole and the ground plate.

While the upper surface of the tibia and fibula was fixed and restrained, the ground plate could move in a vertical direction and simulate the GRF loading. The ground plate was set to come into contact with the outsole first (GRF = 0) and then start to simulate in two steps. During the first step, the Achilles tendon force (Force_AT_) was set to be applied at the instant of ground plate contact and increase until 1/4 of the participant’s weight was applied, after exactly 1 s. For the second step, while the tendon force stayed constant, the ground plate started to move upward and the GRF loading increased. Once the vertical GRF reached half of the participant’s weight, the simulation was complete, at which point the plantar pressure data were collected.

### Heel-strike load and boundary conditions

Across-trial averages of the gait parameters of the model participant were used to simulate the heel strike load. In addition, the across-trial average of ankle dorsiflexion moment of the model participant was introduced into the model by applying an equivalent Achilles tendon force (Novacheck, 1998; Morales-Orcajo et al., 2018).

Boundary conditions were specified with respect to model constraint, type of contact, foot strike angle, and ways to apply external forces (Figure 2B). The upper surface of the tibia and fibula was assumed fixed and restrained in this model. “Remote displacement” was applied at the tongue loop of the shoe; this setting allowed for deformation and movement of the shoe materials around the tongue loop. Similar to the static stand model, there was a bonded contact between the insole and midsole and between the midsole and outsole; frictional contact (μ = 0.6) was used between the foot with the insole and upper and between the outsole and the ground plate. The ground plate was set to form the initial foot strike angle (i.e., obtained from the average running trials) and came into contact with the outsole first and then the external force was loaded in steps. In the first step, the net ankle joint force (Force_Joint_) was applied to the top of the talus for 1 s and then maintained for 3 s to simulate the landing inertia force. For the second step, the Achilles tendon force was applied for 1–2 s and then maintained for 3 s. For the third step, the continuous displacement load was applied through the plate for 2–3 s until the force on the plate reached the same GRF value obtained from the trials. Nonlinear large deformation quasi-static contact analysis was employed in this model.

### Parametric analysis by the Taguchi method

There were four design factors: the midsole hardness (M, 3 levels), the insole material (I, 2 levels), the insole thickness (Fontanella et al., 2013) (T, 3 levels), and a conforming heel-cup (Goske et al., 2006) (H, 3 levels). Detailed combinations are listed in Table 2, demonstrating a total of 54 possible combinations.

**TABLE 2:** Description of design factors and levels related to the Taguchi Method.

Factor	Description	LEVEL1	LEVEL2	LEVEL3
I	Insole material	EVA	Latex	-
T	Insole thickness	0 *mm*	3 *mm*	6 *mm*
H	Heel-cup conforming	0	50% conforming	100% conforming
M	Midsole hardness	Asker C-45	Asker C-60	Asker C-70

We used the statistics-based Taguchi method to optimize design parameters with a reduced number of simulations. We conducted 18 simulations to calculate the effect of design factors based on the orthogonal array L_18_ (2^1^ × 3^3^) generated by Minitab (Version 17.0, Minitab, LLC, State College, PA, United States). After that, if the optimal design combination was not present within the 18 simulations, a further simulation was conducted to perform the best cushioning combination in this study. Thus, the peak pressure of the rearfoot was predicted through at least 18 FE analyses. The mean effect of the respective level of the four design factors on the mechanical responses was computed using Minitab. Considering that our goal was reduced plantar pressure, Taguchi’s “the smaller the better” signal-to-noise (S/N ratio) calculation was employed to measure simulation outcome. An analysis of variance (ANOVA) was performed, which calculated the effect of the design factors to determine the sensitivity of the respective design parameters (Rao et al., 2008; Peng et al., 2022). For the interpretation of statistical analyses, *p* < 0.05 indicates a statistically significant difference.

## Results

### Boundary condition results from running trials

The results of five running trials were analyzed and averaged: GRF was 1149 N, foot strike angle was 14°, Force_Joint_ was -802 N, and ankle dorsiflexion moment was 10 N/m. After dividing the moment by the arm of the Achilles tendon, Force_AT_ was –186 N.

### Experimental validation

**FIGURE 3:**
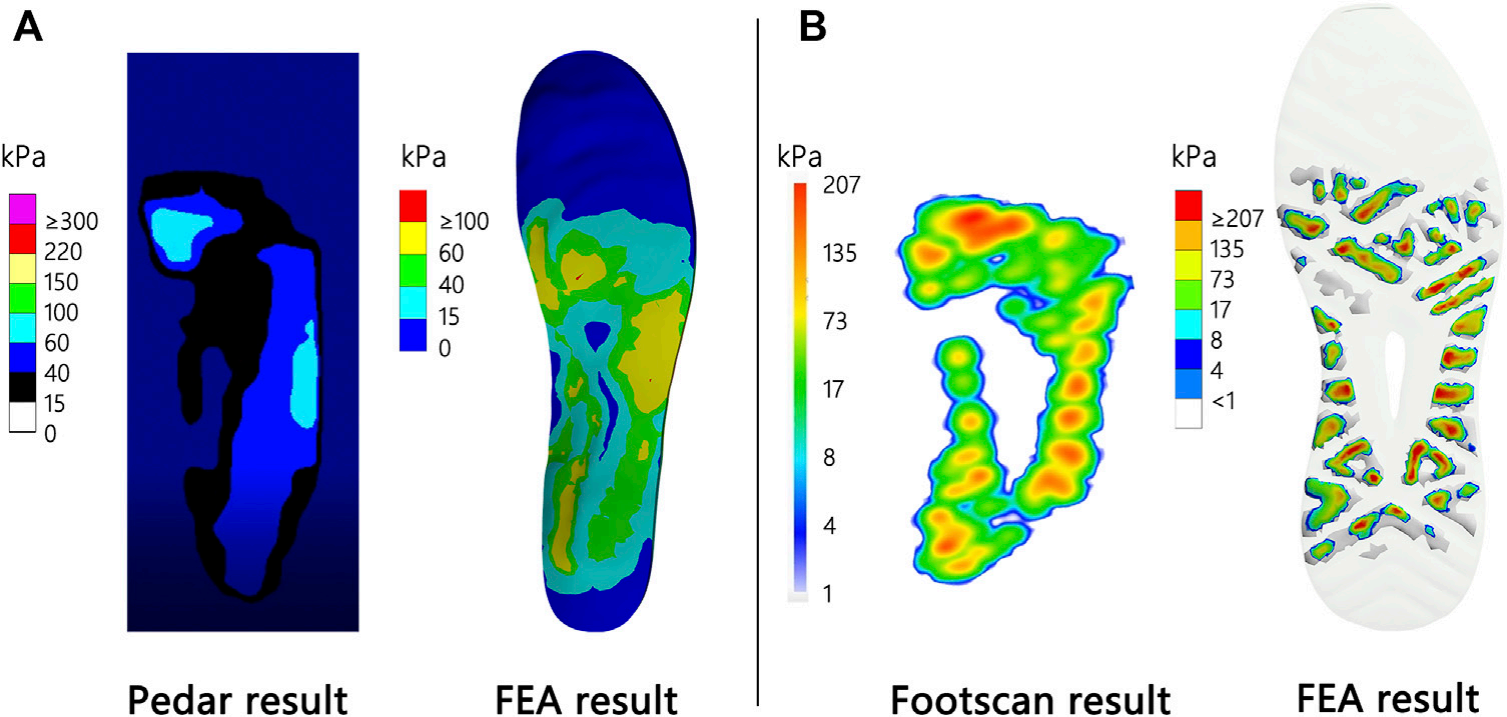
Measured and FE analyzed results of **(A)** insole and **(B)** outsole pressures.

Figure 3 shows the plantar and outsole pressure results with pressure systems and the results predicted with the FE model.

As depicted in Figure 3A, the peak plantar pressure was 95 kPa, i.e., 5 kPa less than the FE result. As revealed by the results, the main pressure positions of the plantar were distributed at the 1st and 2nd metatarsophalangeal joints and the lateral longitudinal arch of the foot.

As depicted in Figure 3B, the peak of plantar pressure was 207 kPa, i.e., 9 kPa less than the FE result. From the distribution results, the main pressure positions of the outsole were distributed at the heel, metatarsal joint, and lateral longitudinal arch of the foot.

### Plantar pressure

**FIGURE 4:**
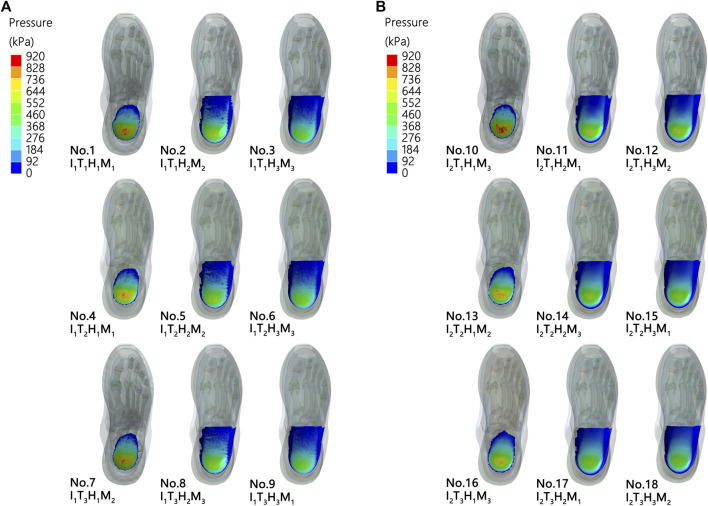
Heel pressure distribution with different shoe designs **(A,B)**.

The results of the FE analyses for predicting the heel pressure distribution during landing are listed in Figure 4 and Table 3. The lowest peak pressure among the 18 simulations was simulation no. 18 (509.10 kPa, I_2_T_3_H_3_M_1_) and the highest was in simulation no. 7 (911.61 kPa, I_2_T_3_H_3_M_1_).

**TABLE 3:** Peak signal-to-noise ratio results of the plantar pressure at heel.

No.	I	T (mm)	H	M	Plantar pressure	
					Peak force (kPa)	S/N ratio (*dB*)
1	EVA	0	0	Asker C-45	802.19	–58.09
2	EVA	0	50%	Asker C-60	811.74	–58.19
3	EVA	0	100%	Asker C-70	729.20	–57.27
4	EVA	3	0	Asker C-45	869.55	–58.79
5	EVA	3	50%	Asker C-60	774.98	–57.79
6	EVA	3	100%	Asker C-70	691.90	–56.80
7	EVA	6	0	Asker C-60	911.61	–59.20
8	EVA	6	50%	Asker C-70	757.36	–57.59
9	EVA	6	100%	Asker C-45	702.20	–56.93
10	Latex	0	0	Asker C-70	905.29	–59.14
11	Latex	0	50%	Asker C-45	623.22	–55.89
12	Latex	0	100%	Asker C-60	615.45	–55.78
13	Latex	3	0	Asker C-60	811.94	–58.19
14	Latex	3	50%	Asker C-70	600.34	–55.57
15	Latex	3	100%	Asker C-45	530.44	–54.49
16	Latex	6	0	Asker C-70	836.86	–58.45
17	Latex	6	50%	Asker C-45	519.73	–54.32
18	Latex	6	100%	Asker C-60	509.10	–54.14

### Taguchi method result

The signal-to-noise ratio results of plantar pressure are presented in Table 3. The highest signal-to-noise ratio among the 18 simulations was in simulation no. 18 (–54.14 *dB*, I_2_T_3_H_3_M_1_) and the lowest was in simulation no. 7 (–59.20 *dB*, I_2_T_3_H_3_M_1_).

Among the four design factors, the use of a conforming heel-cup was identified as the critical design factor for peak heel pressure reduction (53.18%, *p* < 0.001). The insole material was the second most important factor for peak heel reduction (25.89%, *p* = 0.001). The descending order of percentage contribution was conforming heel-cup, insole material, midsole material, and insole thickness (Table 4).

**TABLE 4:** Analysis of variance (ANOVA) of the main contributors for plantar pressure.

Factor	Sum of squares	Degrees of freedom	Mean squares	F-test	*p* value	Contribution percentage (%)
I	11.97	1	11.97	24.81	0.001	25.89
T	1.24	2	0.62	1.29	0.318	2.69
H	24.59	2	12.30	25.48	0.000	53.18
M	3.61	2	1.81	3.74	0.061	7.81
Error	4.83	10	0.48			
Total	46.24	17				

**FIGURE 5:**
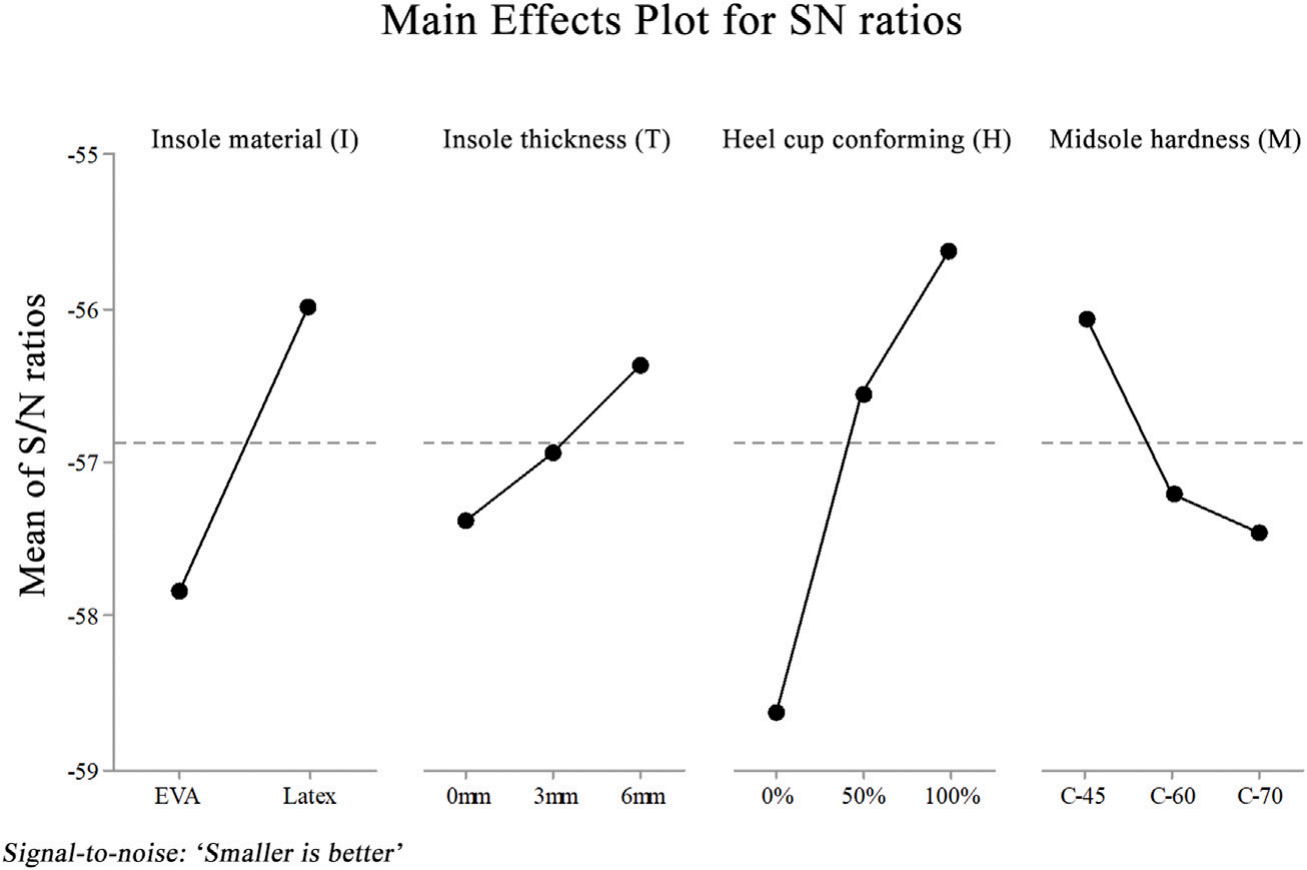
S/N graph for peak pressure. The X axis shows the different levels of each design factor. The Y axis shows the values of the S/N ratio.

The main effect plot further indicates the respective effects of each design factor on reducing heel pressure (Figure 5).

### Optimal combination

**FIGURE 6:**
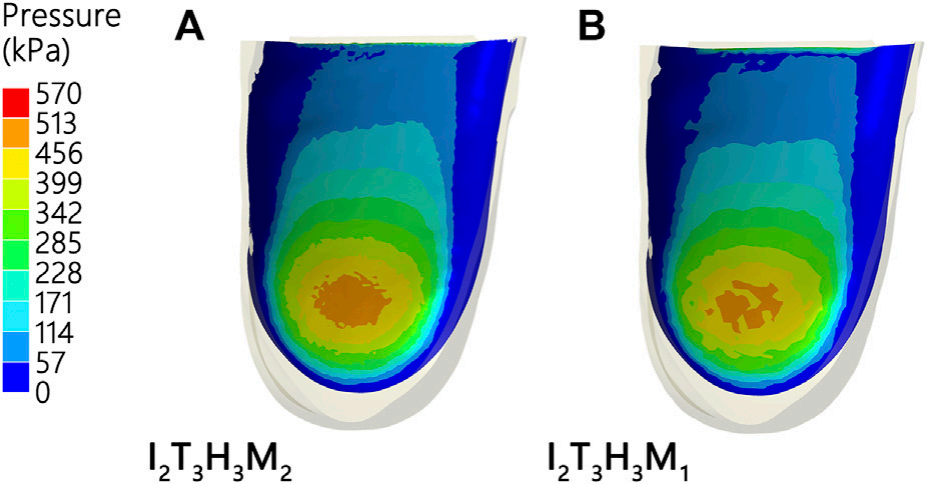
Verification experiment. **(A)**. No. 18 result and**(B)**. optimum result.

An optimal combination to reach the lowest planter pressure was predicted based on the Taguchi method, which consisted of I_2_T_3_H_3_M_1_ (Figure 6).

Table 5 presents a comparison of no. 18 (i.e., the best combination among the 18 simulations) with the optimal design result, which shows that the pressure for the optimal combination is a 9-kPa lower heel pressure than that in the no. 18 combination. Good agreement was observed between the prediction and the FE result under the optimal design, revealing that the peak force is lower than any of the peak force data presented in Table 3.

**TABLE 5:** Verification experiment results.

	No.18 result	Optimal design		Improved value	Improved rate (%)
		Prediction	FE result		
Peak force (kPa)	509.1	501.43	499.89	9.21	1.8
S/N ratio (dB)	–54.14	–54.00	–53.98	0.16	0.3

## Discussion

This study investigated the influence of four factors of running shoe design in reducing plantar pressure during heel-strike running. The study adds to the current body of literature of FE analysis on the impact during running through a complete foot–shoe model instead of a partial model, as well as the testing of shoe parameters with the Taguchi method. The first hypothesis, that all design parameters significantly lower heel pressure, was partially supported, as only the conforming heel-cup and insole material were significant. The second hypothesis, that there is an optimal design to minimize peak heel pressure, was supported. The latex material, 6 mm insole, 100% conforming heel-cup, and midsole hardness of type Asker C-45 were identified as the optimal combination that maximally enhanced the cushioning ability upon heel landing. These findings contribute to a better and more efficient shoe design to reduce the risk of running injuries.

The comparison of FE-predicted pressure and experiment measurements during standing or walking was broadly used in foot finite model validation (Cheung and Zhang, 2008; Hsu et al., 2008; Yu et al., 2008; Yu et al., 2016; Li et al., 2018; Peng et al., 2021a). Our results of validation indicated that the FE-predicted pressure and force distribution have good agreement with results from the Pedar and the Footscan system. Our FE-predicted pressure was 4.3 and 5.3% higher than the Pedar and Footscan system, respectively. The error was less than 10% and acceptable (Zhang et al., 2020), explained by the fact that the higher number of nodes used in the FE model than the number of sensors in experimental measurements may increase the pressure result (Hsu et al., 2008; Yu et al., 2008; Li et al., 2018). Moreover, the optimum combination result from the Taguchi method showed a minimal difference to the FE-predicted results (0.3–1.8%; Table 5), indicating no hidden interaction effects in the FE model. Taken together, the foot–shoe FE model was considered accurate enough to investigate the design feature combinations.

We observed different effects of shoe design factors on peak heel pressure. In the descending order of effect were the conforming heel-cup, insole material, midsole hardness, and insole thickness. The conforming heel-cup result is consistent with previous studies (Bus et al., 2004; Tsung et al., 2004; Cheung and Zhang, 2008). The predicted results revealed that the conforming heel-cup achieved the highest contribution rate (53.18%) among the four design factors in the peak plantar pressure during heel contact. Furthermore, it significantly reduced the peak plantar pressure (*p* < 0.001). The 100% conforming heel-cup maximized the heel contact area and limited the deformation of plantar soft tissue (Chen et al., 2003; Goske et al., 2006) to achieve the effective dispersion of stress. The softer insole material significantly reduced the peak plantar pressure (*p* = 0.001), consistently with other studies using the static stand model (Goske et al., 2006; Cheung and Zhang, 2008; Luo et al., 2011). The insole material had a secondary contribution (25.89%) to the change in the peak plantar pressure, and the application of latex could significantly reduce the excessive heel pressure to a greater extent than does EVA, the latter being commonly used in various orthopedic insoles owing to its good deformability. The possible reason is that the use of latex materials significantly increases the strain of the insole and heel-cup when impacted. Specifically, the large strain of latex material increases the deceleration distance (Clarke et al., 1983; Kaelin et al., 1985; Bennrtt and Ker, 1990); the insole and the heel-cup together constitute a shock absorption buffer of nonlinear elastic material.

The result of midsole hardness was consistent with the thickness of the insole. Although neither of the parameters was significant, trends were observed. The contribution rate of the midsole hardness was 7.81% (*p* = 0.061). The softer the midsole, the lower the plantar pressure, as the former yields greater deformation to reduce the impact of ground reaction forces on the plantar. At present, the midsole hardness of running shoes on the market has usually been from type Asker C-38 to Asker C-70 (Table 6). Thus, changing the hardness has a limited effect on plantar pressure. More elastic midsole material under the same hardness must be considered by shoe manufacturers. The thicker the insole, the lower the plantar pressure (Birke et al., 1994; Linge and Klenerman, 1996; Cheung and Zhang, 2008). In contrast, no significant difference was observed (*p* = 0.318), and the lowest contribution (2.69%) was identified. Due to the application of the reverse engineering shoe model, the choice of insole thickness is limited, giving the insole thickness a smaller role in reducing plantar pressure than previously expected (Chatzistergos et al., 2015).

**TABLE 6:** Hardness characteristics of midsole materials of some sport shoes (Fu et al., 2021).

Brand	Type	Midsole material	Asker C hardness in rearfoot
Do-win	9110	EVA Foam	50–51
Do-win	9111	EVA Foam	55
Do-win	9201	EVA Foam	56
Adidas	Adizero takumi sen boost	TPU Foam	60–67
Adidas	Adizero tempo boost	TPU Foam	38–45
Adidas	Adios 3	TPU Foam	45
Saucony	Type a	EVA Foam	60–68
Saucony	Lexicon 2	EVA Foam	55
Skechers	Go run ultra	EVA Hybrid	45–55
Xtep	160 × 2.0	Nylon Foam	50

The optimal design combination obtained by the FE analysis was compared with the predicted value of the minimum plantar pressure in 18 combinations (Table 5; Figure 6). The peak plantar pressure (1.8%) and S/N ratio (0.3%) in the optimal design combination were reduced as compared with the no. 18 result. The FE results of the peak plantar pressure (Error: 1.54 kPa, 0.31%) and S/N ratio (Error: 0.02 dB, 0.03%) of the optimal design combination were basically consistent with the results predicted using the Taguchi method. We therefore suggest that the design factors of running shoes selected in this study are effective as well as the optimal design combination model calculated by the Taguchi method. The optimal design combination obtained by the Taguchi method significantly reduces the peak plantar pressure of heel landing in the selection of design parameters of running shoes.

The heel-cup conforming design is considered the critical factor for the reduction of plantar pressure, followed by the insole material. These two factors pertain to additional modifications that do not alter the structure of running shoes. The development of customized heel-cups and the selection of different insole materials are effective in the reducing peak plantar pressure during running. Compared to shoe midsole hardness and running surface modifications, a heel-cup conforming design is more cost- and time-efficient for reducing the damage caused by the repeated impact on the heel. For shoe manufacturers, modifying the midsole hardness and the insole thickness brings limited advantages, whereas this situation can be changed by replacing EVA materials with more elastic ones.

Our study has several limitations. The first limitation is the single test subject design, which limits the generalization of results. Considering the complexity of the shoe FE model, single subject studies are often used in existing foot-related FE studies (Chatzistergos et al., 2015; Wen-Ming et al., 2015; Hannah et al., 2016; Van Waerbeke et al., 2022), especially those involving the Taguchi method (Cheung and Zhang, 2008; Zhang et al., 2020; Anggoro et al., 2021; Peng et al., 2022). In the present study, we used a classical static standing FE model to verify the effectiveness of the model. A Taguchi verification experiment (Rao et al., 2008) was conducted to assess optimal combinations. Moreover, the high number of foot FE elements and the complete foot–shoe model contributed to model fidelity, which helps address external validity issues (Wong et al., 2021; Peng et al., 2022). The second limitation is that a restricted number of midsole factors were considered in this study. Different midsole materials, such as the carbon fiber insert and midsole structure (e.g., Nike Vaporfly 4% (Burns and Tam, 2020)), are available on the market. Future research must further consider these factors to accommodate changes in the design and function of running shoes. The third limitation is that our study simplified plantar fascia as five tension-only springs. Compared to studies that built the geometry of the plantar fascia (Peng et al., 2022; Van Waerbeke et al., 2022) and that considered the interaction between plantar fascia and the encapsulated soft tissue (Peng et al., 2021b), our FE model could only be applied and sustained load in the axial direction but not in the cross-sectional direction that may underestimate the stress of the fascia and the insertion points. The last limitation is that our study uses a quasi-static FE model, which is easier than a dynamic FE model, which could also account for the inertia, loading rate, viscoelasticity of materials, etc. Dynamic FE models have been exploited to investigate foot trauma (Shin et al., 2012; Wong et al., 2016; Chen et al., 2019). Due to the complexity of dynamic FE modeling and the multi-models needed to be constructed with the Taguchi method, rarely do FE studies consider both dynamic modeling and the Taguchi method. Future studies should consider discovering the relationship between shoe design factors and cushion effect from heel contact until toe off, which will result in the generation of shoe designs suitable for recommendation to shoe manufacturers.

## Conclusion

To enhance the cushioning effect of running shoes during heel landing, a better conforming heel-cup and a softer insole should be considered. The results from this study suggest that runners who want to relieve plantar pressure should consider a custom insole with a conforming heel-cup.

## Data Availability

The raw data supporting the conclusion of this article will be made available by the authors without undue reservation.
